# Comparison of pulmonary vein isolation using cryoballoon, high-power short-duration, and conventional radiofrequency ablation for atrial fibrillation: a propensity score-weighted study

**DOI:** 10.3389/fcvm.2023.1238363

**Published:** 2023-10-09

**Authors:** Hanjin Park, Je-Wook Park, Daehoon Kim, Hee Tae Yu, Tae-Hoon Kim, Jae-Sun Uhm, Boyoung Joung, Moon-Hyoung Lee, Chun Hwang, Hui-Nam Pak

**Affiliations:** Division of Cardiology, Department of Internal Medicine, Yonsei University College of Medicine, Yonsei University Health System, Seoul, Republic of Korea

**Keywords:** atrial fibrillation, catheter ablation, cryoballoon ablation, high-power short-duration ablation, heart rate variability

## Abstract

**Background:**

The comparative efficacy, saftey, and heart rate variability (HRV) parameters after pulmonary vein isolation using cryoballoon (Cryo-PVI), high-power short-duration (HPSD-PVI), and conventional radiofrequency ablation (conventional-PVI) for atrial fibrillation (AF) is unclear.

**Materials and methods:**

In this propensity score-weighted, retrospective analysis of a single-center cohort, we analyzed 3,395 patients (26.2% female, 74.5% paroxysmal AF) who underwent AF catheter ablation without an empirical left atrial ablation. Procedural factors, recurrence rates, complication rates, and the post-procedural HRV parameters were compared across the Cryo-PVI (*n* = 625), HPSD-PVI (*n* = 748), and conventional-PVI (*n* = 2,022) groups.

**Results:**

Despite the shortest procedural time in the Cryo-PVI group (74 min for Cryo-PVI vs. 104 min for HPSD-PVI vs. 153 min for conventional-PVI, *p* < 0.001), the major complication (*p* = 0.906) and clinical recurrence rates were similar across the three ablation groups (weighted log-rank, *p* = 0.824). However, the Cryo-PVI group was associated with a significantly lower risk of recurrent AF in patients with paroxysmal AF [weighted hazard ratio (WHR) 0.57, 95% confidence interval (CI) 0.37–0.86], whereas it was associated with a higher risk of recurrent AF in patients with persistent AF (WHR 1.41, 95% CI 1.06–1.89, *p* for interaction of <0.001) compared with the conventional-PVI group. In the subgroup analysis for the HRV, the Cryo-PVI group had the highest low-frequency-to-high-frequency ratio at 1-year post-procedure, whereas the HPSD-PVI group had the lowest low-frequency-to-high-frequency ratio at 1-year post-procedure (*p* < 0.001).

**Conclusions:**

The Cryo-PVI group had better rhythm outcomes in patients with paroxysmal AF but worse rhythm outcomes in patients with persistent AF and a higher long-term post-procedural sympathetic nervous activity and sympatho-vagal balance compared with the conventional-PVI group.

## Introduction

Atrial fibrillation (AF) is a growing global health burden that is associated with substantial morbidity and mortality (1). Rhythm control for AF improves cardiovascular outcomes (2, 3), and the need for AF catheter ablation (AFCA) is continuously increasing. However, long-term maintenance of sinus rhythm after conventional pulmonary vein isolation (conventional-PVI) has been unsatisfactory (4) and has been limited by frequent pulmonary vein (PV) reconnections due to tissue edema and non-transmural lesion formation (5, 6). Also, conventional-PVI is technically complicated, and the operator’s skills and experiences are important for an efficient and safe procedure. Therefore, alternative ablation technologies have gained interest to improve clinical rhythm outcomes, shorten procedural times, and reduce procedure-related complications in patients with AF.

Recently, high-power short-duration pulmonary vein isolation (HPSD-PVI) has gained interest as an alternative ablation strategy that uses resistive heating with better transmural lesion formation (7, 8). Kottmaier et al. (9) reported that HPSD-PVI (70 W for 5–7 s) improves rhythm outcomes compared with conventional-PVI in patients with paroxysmal AF (PAF). Another promising technology, cryoballoon pulmonary vein isolation (Cryo-PVI), freezes and ablates large volumes of PV ostium at a single shot with less catheter manipulation and is less sensitive to the operator's experiences compared with conventional-PVI. Cryo-PVI showed comparable rhythm outcomes with conventional-PVI among patients with PAF, and a rather low PV reconnection rate has been reported (10–12).

However, studies that directly compare the three ablation modalities (conventional-PVI, HPSD-PVI, and Cryo-PVI) are limited. In this propensity score-weighted study, we compared the efficacy and safety outcomes along with changes in heart rate variability (HRV) parameters after Cryo-PVI, HPSD-PVI, and conventional-PVI in patients with AF.

## Materials and methods

### Study population

**Figure 1:**
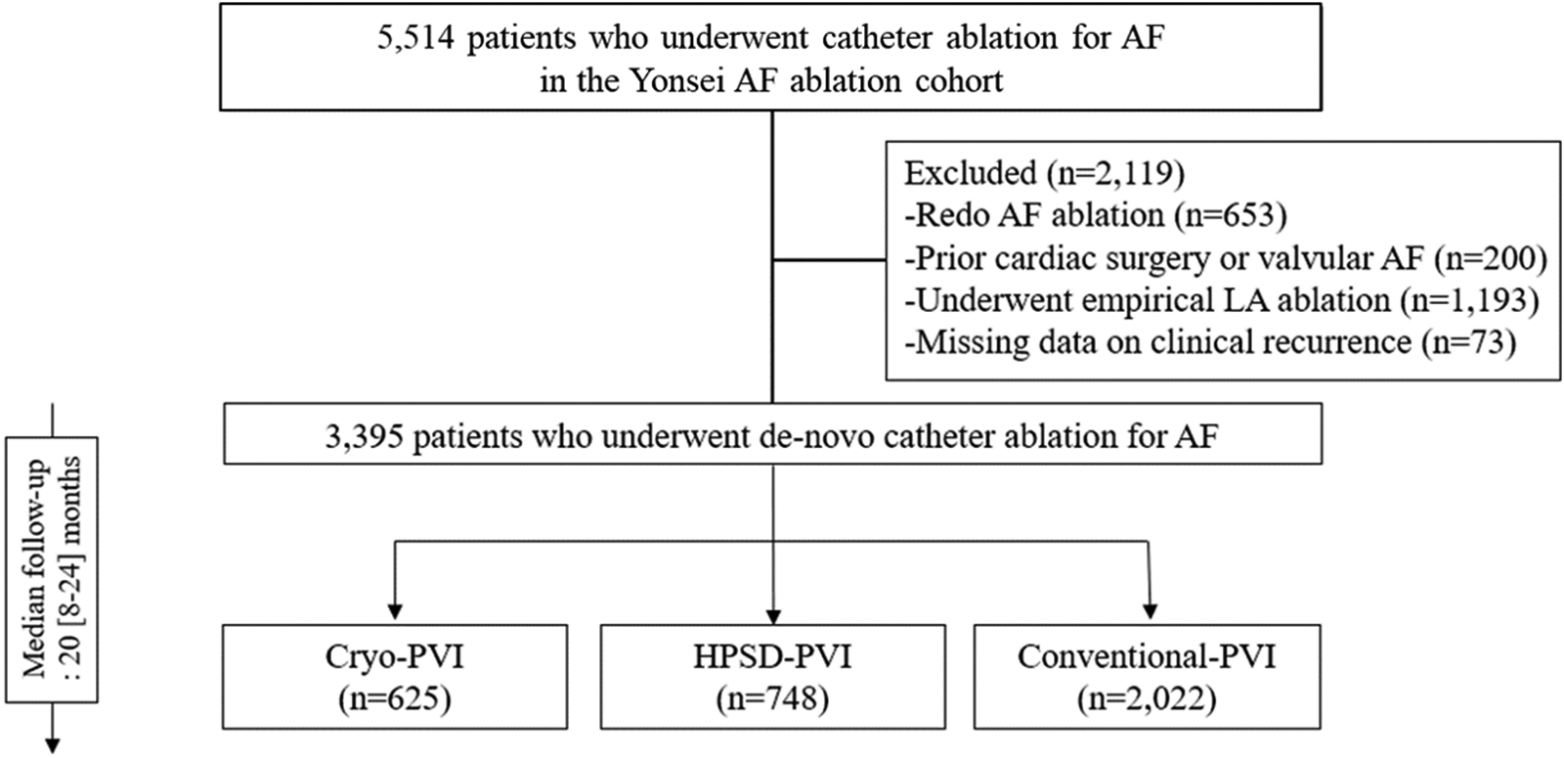
Study flow chart. AAD, anti-arrhythmic drugs; AF, atrial fibrillation; AFCA, atrial fibrillation catheter ablation; ASD, absolute standardized difference; CPVI, circumferential pulmonary vein isolation; E/e’, ratio of early diastolic mitral inflow velocity to early diastolic mitral annulus velocity; HPSD, high-power short-duration; IPTW, inverse probability of treatment weighting; LA, left atrium; LV, left ventricle; LVEDD, left ventricular end diastolic dimension; PVI, pulmonary vein isolation; SVC-RA, superior pulmonary vein-right atrial.

The study protocol was approved by the Institutional Review Board of the Yonsei University Health System. It adhered to the principles of the Declaration of Helsinki. A written informed consent was obtained from each patient in the Yonsei AF ablation cohort. We included 3,395 participants who underwent *de novo* AFCA without an empirical left atrial (LA) ablation (Figure 1). The exclusion criteria of this study were identified, namely, (1) prior history of AF ablation, (2) AF with rheumatic valvular disease, (3) prior cardiac surgery, and (4) extra-PV LA ablation. The participants were categorized into three groups according to the AFCA modality: Cryo-PVI (*n* = 625), HPSD-PVI (*n* = 748), and conventional-PVI (*n* = 2,022).

### Echocardiographic evaluation

All participants underwent pre-procedural transthoracic echocardiography. The LA volume index (LAVI), LV end-diastolic dimension (LVEDD), LV ejection fraction, and peak trans-mitral inflow velocity to tissue Doppler echocardiography of the peak septal mitral annular velocity (E/e') were assessed in accordance with the guidelines published by the American Society of Echocardiography (13).

### Electrophysiological mapping and AFCA

Prucka CardioLab Electrophysiology system (General Electric Medical Systems, Inc., Milwaukee, WI, USA) was used for intracardiac electrogram recordings. After a trans-septal puncture, a multi-view pulmonary venogram was obtained, and 350–400 s of activated clotting time was maintained via intravenous heparin injection. A 3D electro-anatomical mapping system (NavX; Abbott or CARTO; Biosense Webster) merged with 3D spiral CT was used to guide the AFCA in all patients. The luminal esophageal temperature was monitored and maintained below 38.4°C for the HPSD-PVI or conventional-PVI and 15°C for the Cryo-PVI. The esophageal temperature in most of the conventional-PVI group was not monitored because routine esophageal temperature monitoring started in 2019.

For the HPSD-PVI, we used an open irrigated-tip deflectable catheter (FlexAbility; Abbott Inc.). Radiofrequency (RF) power of 50–60 W for 7–15 s and 50 W for <10 s was used for the anterior and posterior area of the LA with a target temperature of 45°C. For the conventional-PVI, we used an open irrigated, 3.5-mm-tip deflectable catheter (Celsius and Smart-Touch; Johnson & Johnson, Inc., Diamond Bar, CA, USA; Coolflex and FlexAbility, Abbott Inc., Minnetonka, MN, USA). RF power of 30–35 and 20–25 W was used for the anterior and posterior area of the LA, respectively. Ablation of the cavotricuspid isthmus (CTI) was performed in most of the patients in the HPSD-PVI and conventional-PVI groups except for those with AV conduction disease. All patients underwent CPVI that encircled the right and left PVs.

For the Cryo-PVI group, a 28-mm cryoballoon (Arctic Front Advance, Medtronic) with a 12 Fr deflectable trans-septal sheath (FlexCath Advance, Medtronic) was used with a multipolar spiral catheter (Achieve, 20 mm; Medtronic). Optimal PV ostium and cryoballoon contact (PV occlusion) was confirmed using a contrast medium. Each PV ostium was frozen for 4 min. The protocol for cryoballoon dosing was modified from the time-to-isolation-based ICE-T (Individualized Cryoballoon Energy Pulmonary Vein Isolation Guided by Real-Time Pulmonary Vein Recordings) protocol (14). The freezing was discontinued, and the catheter was repositioned when the time to isolation exceeded 90 s to achieve better PV occlusion.

### Post-ablation management and follow-up

Telemetry monitoring was used in all patients before discharge. Except for those who had extra-PV triggers, symptomatic frequent premature atrial complexes (PACs), non-sustained atrial tachycardia (AT), or AF, all patients were discharged without anti-arrhythmic drugs (AADs). Patients were regularly followed up at the outpatient clinic (1, 3, 6, and 12 months from discharge and every 6 months thereafter) or whenever they experienced signs and symptoms suggestive of arrhythmia recurrence. A 12-lead ECG was obtained at every outpatient clinic visit with regular 24-h Holter recordings (3 and 6 months from discharge and every 6 months thereafter) in accordance with the guidelines issued by the 2017 Heart Rhythm Society/European Heart Rhythm Association/European Cardiac Arrhythmia Society Expert Consensus Statement (15). An 24-h Holter monitoring or event monitor recordings were performed in patients who had signs or symptoms of arrhythmia recurrence. All 24-h Holter recordings were analyzed and verified by an independent researcher.

AF recurrence was defined as any ECG documentation of AF or AT of at least 30 s. Early recurrence was defined as AF recurrence of ≤3 months post-procedure, whereas a clinical recurrence was defined as AF recurrence of >3 months post-procedure.

### Holter monitor recordings and the heart rate variability analysis

The HRV parameters were obtained from the 24-h Holter monitor recordings with a GE Marquette MARS 8000 Holter analyzer (General Electric Medical System, Inc.). Premature ventricular complexes, PACs, electrical artifacts, or low-quality recordings were excluded from the analysis. All 24-h Holter recordings were reviewed by an experienced operator. We were not able to obtain HRV parameters from all participants, and a total of 73 of the Cryo-PVI group, 136 of the HPSD-PVI group, and 1,049 of the conventional-PVI group had serial long-term HRV parameter follow-up data at pre-ablation and 3-month and 1-year post-ablation.

As a sensitivity analysis, we provided the HRV results for a total of 1,529, 1,809, 1,464, and 637 individuals who had available HRV data (but without complete serial follow-up data) at pre-ablation and 3-month, 1-year, and 2-year post-ablation periods, respectively. The time-domain HRV parameters analyzed were mean heart rate (HR) and root mean square of the differences between successive NN intervals (rMSSD). The frequency-domain HRV parameters analyzed were low-frequency component (LF; 0.040 Hz–0.150 Hz), high-frequency component (HF; 0.150 Hz–0.400 Hz), and the LF/HF ratio. In brief, the mean HR is a surrogate of the HRV, and rMSSD represents the beat-to-beat variance in the heart rate in which low mean HR and high rMSSD indicate an increased HRV (16). The HF and LF component represents parasympathetic and sympathetic nervous activity, and the LF/HF ratio represents sympatho-vagal balance (17).

### Statistical analysis

Categorical variables were reported as numbers (percentages) and were compared using the Chi-square or Fisher's exact test. Continuous variables with normal distribution were reported as mean ± standard deviation and non-normal distribution as medians (interquartile range), in which normality was tested using the Shapiro–Wilk or Kolmogorov–Smirnov test. Continuous variables were compared with using the ANOVA test (normal distribution) or the Kruskal–Wallis test (non-normal distribution).

Among the echocardiographic parameters, 121 missing values for E/e' (10 in Cryo-PVI, 15 in HPSD-PVI, 96 in conventional-PVI), 65 for LAVI (eight in Cryo-PVI, 12 in HPSD-PVI, 45 in conventional-PVI), and 75 for LVEDD (seven in Cryo-PVI, 14 in HPSD-PVI, 54 in conventional-PVI) in our study sample were reported. We used multiple imputation by chain equation using mice package in R statistics to generate 50 matrices of imputed dataset. After then, for an unbiased comparison between the AFCA groups, we used an inverse probability of treatment weighting (IPTW) approach. The propensity score, which is the probability of receiving treatment, was estimated using a multinomial logistic regression based on sociodemographic factors, medical and medication history, and echocardiographic parameters (variables are presented in Table 1). We examined the balance between the AFCA groups using the absolute standardized differences of all covariates with a threshold of 0.1 to indicate an imbalance. The weights were truncated at the 1st and 99th percentiles to avoid extreme weights.

**Table 1:** Baseline characteristics before and after inverse probability of treatment weighting.

	Pre-IPTW				Post-IPTW			
	Cryo-PVI	HPSD-PVI	Conventional- PVI	Maximum ASD	Cryo-PVI	HPSD-PVI	Conventional- PVI	Maximum ASD
Total, *n*	625	748	2,022		464^a^	468^a^	1833^a^	
Age, year	63 (55–70)	61 (53–67)	59 (51–66)	0.224	60 (53–67)	60 (52–66)	60 (52–66)	0.046
Male	458 (73.3)	557 (74.5)	1,491 (73.7)	0.018	345 (74.3)	349 (74.5)	1,362 (74.3)	0.004
Height, m	1.69 (1.62–1.74)	1.70 (1.63–1.75)	1.69 (1.62–1.74)	0.088	1.70 (1.63–1.74)	1.69 (1.63–1.75)	1.69 (1.62–1.74)	0.035
Paroxysmal AF	339 (54.2)	471 (63.0)	1,718 (85.0)	0.468	292 (62.9)	328 (70.1)	1,408 (76.8)	0.205
Hypertension	329 (52.6)	339 (45.3)	894 (44.2)	0.113	232 (50.1)	230 (49.2)	836 (45.6)	0.060
Diabetes	99 (15.8)	121 (16.2)	282 (13.9)	0.042	70 (15.0)	73 (15.6)	269 (14.7)	0.016
BMI, kg/m^2^	24.3 (22.7–26.4)	24.8 (23.1–26.8)	24.8 (23.1–26.8)	0.075	24.6 (23.0–26.5)	24.8 (23.0–26.6)	24.8 (23.1–26.8)	0.030
Heart failure	100 (16.0)	133 (17.8)	168 (8.3)	0.190	60 (13.0)	61 (13.1)	192 (10.5)	0.052
Vascular disease	34.0 (5.2)	33.0 (4.4)	167 (8.3)	0.106	21 (4.6)	24 (5.1)	134 (7.3)	0.075
CHA_2_DS_2_-VASc	2 (1–3)	1 (1–2)	1 (0–2)	0.172	1 (1–2)	1 (1–2)	1 (0–2)	0.029
H2FPEF score	5 (4–6)	5 (4–6)	5 (4–6)	0.101	5 (4–6)	5 (4–6)	5 (4–6)	0.036
Echocardiographic parameters								
EF, %	64 (60–69)	64 (59–68)	64 (60–69)	0.059	64 (60–69)	64 (59–69)	64 (60–69)	0.014
E/e'	9.0 (7.0–11.0)	9.2 (7.4–11.9)	9.0 (7.0–11.0)	0.074	8.9 (7.2–11.1)	9.1 (7.3–11.9)	9.0 (7.1–11.2)	0.065
LAVI, mL/m^2^	32.3 (26.4–40.0)	37.1 (29.9–45.4)	32.3 (26.4–40.0)	0.278	34.8 (29.2–42.9)	34.7 (28.2–43.0)	33.9 (27.4–41.4)	0.068
LVEDD, mm	50.0 (47.0–53.0)	50.0 (47.0–53.0)	50.0 (47.0–53.0)	0.086	50.0 (46.1–53.0)	50.0 (46.0–53.0)	50.0 (47.0–53.0)	0.029
Procedure characteristics								
CPVI	625 (100.0)	748 (100.0)	2,022 (100.0)	NA	464 (100.0)	468 (100.0)	1,833 (100.0)	NA
Empirical LA ablation	0 (0.0)	0 (0.0)	0 (0.0)	NA	0 (0.0)	0 (0.0)	0 (0.0)	NA
SVC-RA ablation	136 (21.8)	614 (82.1)	935 (46.2)	0.952	128 (27.6)	261 (55.7)	893 (48.7)	0.394
AAD after 3 months	212 (33.9)	253 (33.8)	799 (39.5)	0.079	187 (40.3)	162 (34.6)	698 (38.1)	0.092
Beta-blocker	326 (52.2)	364 (48.7)	797 (39.4)	0.172	225 (48.5)	231 (49.3)	803 (43.8)	0.080

Values are presented as mean ± standard deviation or *n* (%).

AAD, anti-arrhythmic drugs; AF, atrial fibrillation; AFCA, atrial fibrillation catheter ablation; ASD, absolute standardized difference; CPVI, circumferential pulmonary vein isolation; E/e’, ratio of early diastolic mitral inflow velocity to early diastolic mitral annulus velocity; HPSD, high-power short-duration; IPTW, inverse probability of treatment weighting; LA, left atrium; LV, left ventricle; LVEDD, left ventricular end diastolic dimension; PVI, pulmonary vein isolation; SVC-RA, superior pulmonary vein-right atrial.

^a^ Effective sample size.

We conducted a weighted Kaplan–Meier analysis to compare the clinical recurrence rates across the three AFCA groups. In addition, the predictors associated with clinical recurrence were assessed using Cox proportional hazard models.

As the AF type and SVC-RA ablation (among the covariates) were not sufficiently balanced after IPTW (Table 1), the weighted Cox proportional hazard model was additionally adjusted for those covariates (doubly robust IPTW model). The proportional hazard assumption was not violated as examined by Schoenfeld residual plots (18). We performed multiple subgroup analyses using the weighted Cox proportional hazards regression analysis by the pre-specified baseline covariates to detect any potential interaction with clinical recurrence according to the AFCA modality. For the subgroup analysis, a BMI of ≥25 kg/m^2^ was considered obese according to the Asian guidelines on the definition of obesity (19). An LAVI of >34 mL/m^2^ was considered enlarged LA according to the guidelines published by the American Society of Echocardiography (13).

All analyses were performed using R statistics, version 4.0.2 software (R Foundation for Statistical Computing), and a two-sided *p*-value of <0.05 was considered statistically significant.

### Sensitivity analysis

Several sensitivity analyses were conducted in this study. First, because the study sample included patients who underwent conventional-PVI in earlier years compared with Cryo-PVI (started 2019) or HPSD-PVI (started 2018), we repeated the main analysis after excluding patients who underwent conventional-PVI before the year at which first Cryo-PVI or HPSD-PVI was performed. Second, because the study sample included patients who underwent conventional-PVI with the Celsius catheter, the non-contact force electrode catheter, we repeated the main analysis after excluding patients (0.8%, 27 of 3,395) to reduce the risk of potential confounding. Third, we analyzed the risk of clinical recurrence according to 3-month and 1-year post-ablation HRV. The risk of clinical recurrence according to 1-year post-ablation HRV was calculated only for AF recurrences that occurred after 1 year, whereas that according to 3-month post-ablation HRV included all AF recurrences. The weighted hazard ratios per one-standard deviation increase in each of the HRV parameters were assessed with the same doubly robust IPTW model.

## Results

### Baseline characteristics and procedural outcomes

Overall, the study participants had a mean age of 60 (52–67) years, 889 (26.2%) were female, and 2,528 (74.5%) had PAF. The baseline characteristics of the study participants according to the AFCA modality are presented in Table 1. After IPTW, the baseline covariates were well-balanced across the three ablation groups (Table 1) except for AF type and SVC-RA ablation (Supplementary Figures S1, S2).

The ablation characteristics are presented in Table 2. The procedural time was shortest in the Cryo-PVI group (*p* < 0.001 for three groups, Table 2), and early recurrence was lowest in the HPSD-PVI group (*p* = 0.010 for three groups, Table 2). The median time to recurrence across the three AFCA groups was 8.5 months for Cryo-PVI and 8 months for HPSD-PVI and conventional-PVI groups (*p* = 0.032 for three groups, Table 2).

**Table 2:** Procedural and clinical outcomes.

	Pre-IPTW				Post-IPTW			
	Cryo-PVI	HPSD-PVI	Conventional-PVI	*p*-Value	Cryo-PVI	HPSD-PVI	Conventional-PVI	*p*-Value
Total, *n*	625	748	2,022		464^a^	468^a^	1833^a^	
Procedure time, min	72 (61–85)	110 (95–130)	152 (104–182)	<0.001	74 (62–90)	104 (90–123)	153 (105–183)	<0.001
Fluoroscopic time, min	17 (14–22)	23 (18–28)	30 (20–40)	<0.001	18 (14–23)	23 (18–29)	29 (20–40)	<0.001
Ablation time, s	1,146 (963–1,407)	2,045 (1,718–2,522)	3,770 (2,698–4,703)	<0.001	1,165 (963–1,457)	1,918 (1,587–2,400)	3,792 (2,698–4,723)	<0.001
Post-ablation medication								
ARB/ACEi	237 (37.9)	257 (34.4)	632 (31.3)	0.006	163 (35.2)	160 (34.2)	598 (32.6)	0.613
Early recurrence	172 (27.5)	203 (27.1)	606 (30.0)	0.242	130 (28.0)	111 (23.7)	563 (30.7)	0.010
Clinical recurrence	145 (23.4)	141 (18.9)	485 (24.0)	0.015	99 (21.4)	93 (19.9)	449 (24.5)	0.100
Time to clinical recurrence, months	9.0 (5.0–14.0)	8.0 (5.0–14.0)	8.0 (5.0–14.0)	0.021	8.5 (5.0–14.0)	8.0 (5.0–14.0)	8.0 (5.0–14.0)	0.032
Total complication	33 (5.3)	25 (3.3)	80 (4.0)	0.180	21 (4.5)	15 (3.2)	71 (3.9)	0.453
Major complication	20 (3.2)	18 (2.4)	48 (2.4)	0.501	14 (3.0)	13 (2.8)	45 (2.5)	0.906
Tamponade	7 (35.0)	9 (50.0)	21 (43.8)		5 (35.2)	6 (50.0)	20 (46.5)	** **
Hemopericardium	2 (10.0)	4 (22.2)	8 (16.7)		1 (11.7)	3 (23.5)	7 (15.8)	
Phrenic nerve palsy	8 (40.0)	0 (0.0)	2 (4.2)		7 (37.8)	0.0	2 (3.8)	
Pulmonary vein stenosis	1 (5.0)	0 (0.0)	1 (2.1)		0 (4.1)	0.0	1 (2.3)	
SSS or AVB	0 (0.0)	2 (11.1)	4 (8.3)		0 (0.0)	1 (6.2)	3 (6.9)	
AV fistula	2 (10.0)	1 (5.6)	6 (12.5)		1 (11.2)	2 (15.0)	7 (13.9)	
AE fistula	0 (0.0)	0 (0.0)	1 (2.1)		0 (0.0)	0.0	1 (1.9)	
Stroke	0 (0.0)	2 (11.1)	4 (8.3)		0 (0.0)	1 (5.3)	3 (7.1)	
Pseudoaneurysm	0 (0.0)	0 (0.0)	1 (2.1)		0 (0.0)	0.0	1 (2.0)	

Values are presented as median (1st quartile, 3rd quartile) or *n* (%).

ACEi, angiotensin-converting enzyme inhibitor; AE, aortoesophageal; ARB, angiotensin receptor blocker; AV, arteriovenous; AVB, atrioventricular block. Other abbreviations are the same as in Table 1.

^a^ Effective sample size.

The total and major complication rates did not significantly differ across the three ablation groups (*p* = 0.906 for major complications, Table 2). Specifically, among the cardiac tamponade, three from conventional-PVI and one from HPSD-PVI required pericardial window formation, whereas others were managed with pericardiocentesis. All the phrenic nerve palsies after Cryo-PVI were transient, and all the strokes in the conventional-PVI did not result in a clinically meaningful sequelae. One pulmonary vein stenosis from conventional-PVI required a percutaneous stent, and one SSS/AVB from conventional-PVI required a pacemaker implantation.

### Overall rhythm outcome

**Figure 2:**
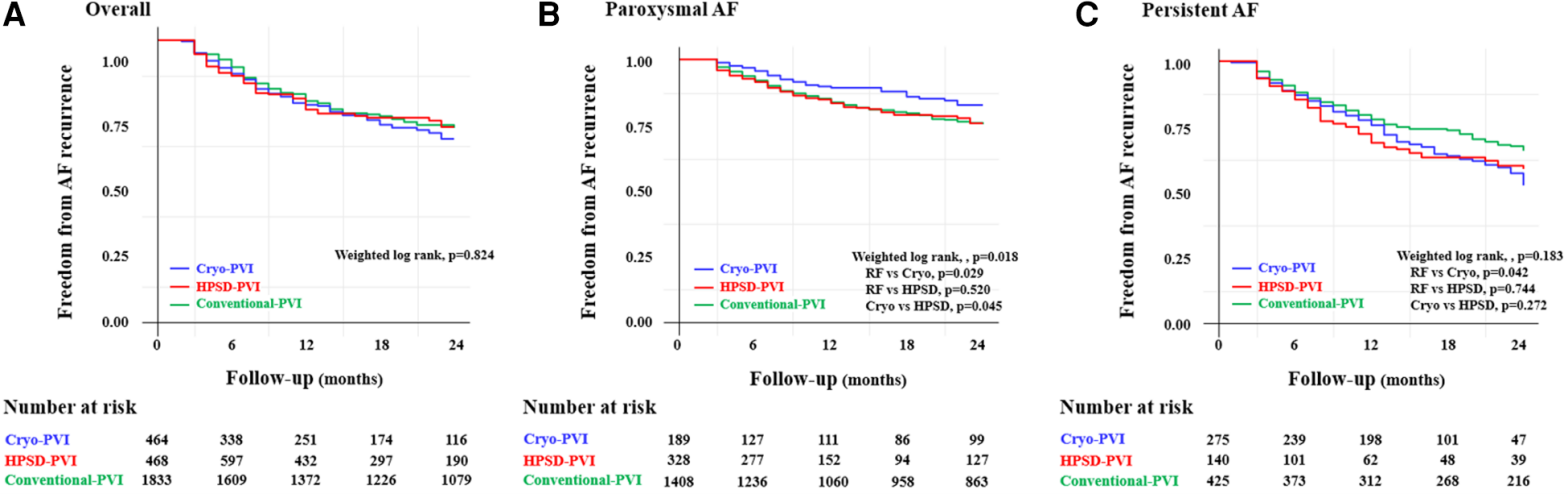
Freedom from clinical recurrence according to the AFCA modality among (**A**) overall, (**B**) paroxysmal AF, and (**C**) persistent AF patients. PaF, paroxysmal atrial fibrillation; PeAF, persistent atrial fibrillation; AAD, anti-arrhythmic drugs; AF, atrial fibrillation; AFCA, atrial fibrillation catheter ablation; ASD, absolute standardized difference; CPVI, circumferential pulmonary vein isolation; E/e’, ratio of early diastolic mitral inflow velocity to early diastolic mitral annulus velocity; HPSD, high-power short-duration; IPTW, inverse probability of treatment weighting; LA, left atrium; LV, left ventricle; LVEDD, left ventricular end diastolic dimension; PVI, pulmonary vein isolation; SVC-RA, superior pulmonary vein-right atrial.

Clinical recurrences within 24 months after AFCA were analyzed in this study because of differences in rhythm follow-up duration across the three ablation groups. For a median of 20 (8–24) months, the overall clinical recurrence rate was similar across the three ablation groups (weighted log-rank, *p* = 0.824 for the three ablation groups, Figure 2A). In the weighted Cox regression analysis, the overall risk of clinical recurrence in the Cryo-PVI or HPSD-PVI group was similar compared with the conventional-PVI group (Supplementary Table S2).

### Subgroup analysis

Among patients with PAF, the Cryo-PVI group had a lower rate of clinical recurrence compared with the conventional-PVI group (weighted log-rank, *p* = 0.018 for the three groups, *p* = 0.029 for the Cryo-PVI vs. conventional-PVI group, Figure 2B). Among patients with persistent AF (PeAF), no significant difference in the rate of clinical recurrence across the three ablation groups (weighted log-rank, *p* = 0.183, Figure 2C) was found. However, the rate of clinical recurrence in the Cryo-PVI group was significantly worse than the rate of clinical recurrence in the conventional-PVI group (weighted log-rank, *p* = 0.042, Figure 2C).

**Figure 3:**
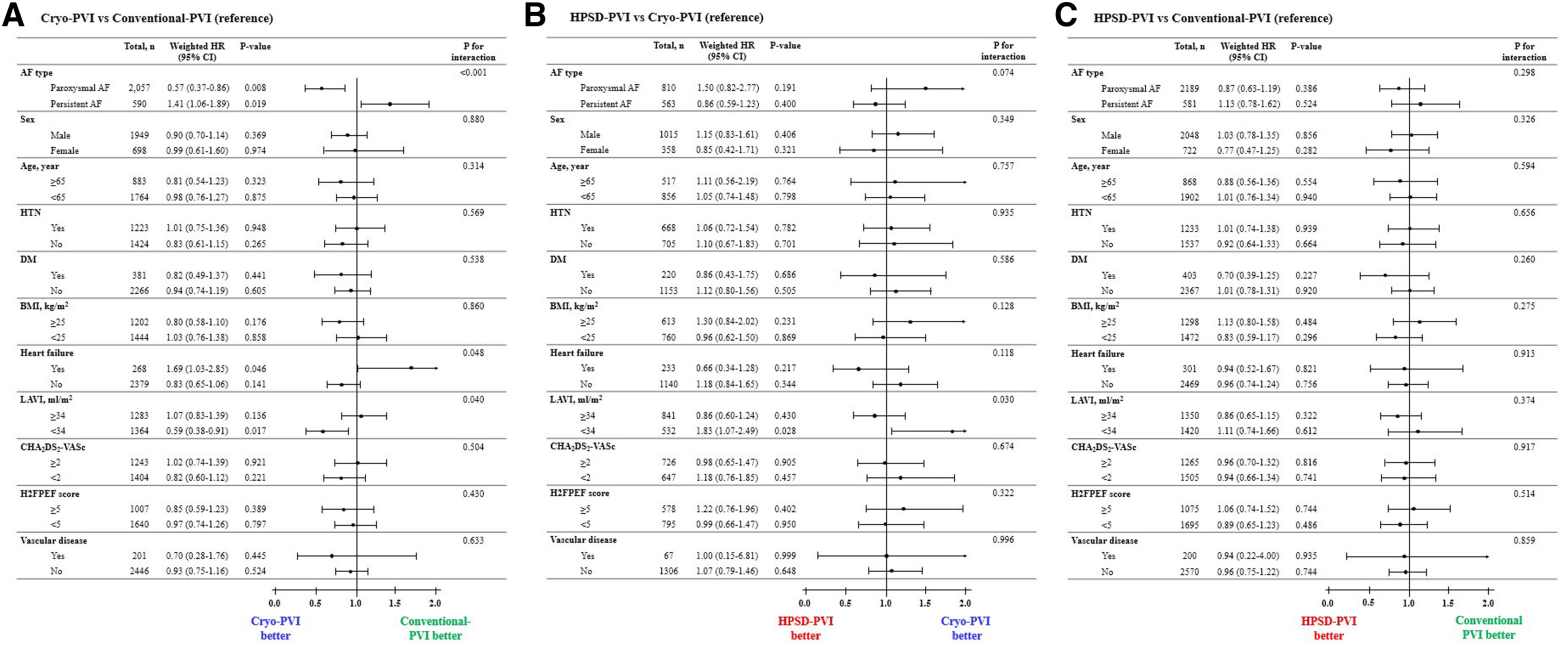
Subgroup analysis of the risk of clinical recurrence according to the AFCA modality: (**A**) Cryo-PVI versus Conventional-PVI, (**B**) HPSD-PVI versus Cryo-PVI, and (**C**) HPSD-PVI versus Conventional-PVI. AAD, anti-arrhythmic drugs; AF, atrial fibrillation; AFCA, atrial fibrillation catheter ablation; ASD, absolute standardized difference; CPVI, circumferential pulmonary vein isolation; E/e’, ratio of early diastolic mitral inflow velocity to early diastolic mitral annulus velocity; HPSD, high-power short-duration; IPTW, inverse probability of treatment weighting; LA, left atrium; LV, left ventricle; LVEDD, left ventricular end diastolic dimension; PVI, pulmonary vein isolation; SVC-RA, superior pulmonary vein-right atrial. ^a^Weighted HR was additionally adjusted for AF type and SVC-RA ablation.

In the subgroup analysis for the weighted hazard ratio, the risk of clinical recurrence in the Cryo-PVI group was independently associated with AF type compared with the conventional-PVI group (Figure 3A). The risk of clinical recurrence in the Cryo-PVI group was significantly lower among patients with PAF [weighted hazard ratio (WHR) 0.57, 95% CI 0.37–0.86, *p* = 0.008, Figure 3A], whereas it higher among patients with PeAF (WHR 1.41, 95% CI 1.06–1.89, *p* = 0.019, Figure 3A) compared with the conventional-PVI group (*p* for interaction of <0.001, Figure 3A). In addition, a significant interaction with clinical recurrence according to LAVI in the conventional-PVI and HPSD-PVI groups was identified compared with the Cryo-PVI group (LAVI; *p* for interaction of 0.040 for the conventional-PVI group and 0.030 for the HPSD-PVI group, Figure 3A,B, respectively). In contrast, no significant interaction with clinical recurrence across the specified subgroups in the HPSD-PVI group was noted compared with the conventional-PVI group (Figure 3C).

The sensitivity analyses after excluding patients who underwent conventional-PVI before the first Cryo-PVI or HPSD-PVI (Supplementary Figure S3) and excluding patients who underwent conventional-PVI with the Celsius catheter (Supplementary Figure S4) showed similar findings with the main analysis representing better rhythm outcomes after Cryo-PVI in patients with PAF and worse rhythm outcomes in patients with PeAF.

### Changes in the HRV parameters after AFCA

The pre-ablation HRV parameters were not significantly different across the three ablation groups (Table 3). However, the post-ablation LF/HF ratio revealed significant differences that persisted for up to 1 year of follow-up. At 1-year post-procedure, the LF/HF ratio was highest in the Cryo-PVI group and lowest in the HPSD-PVI group (*p* < 0.001, Table 3). Similar results were found in the sensitivity analysis among those without full serial HRV follow-up data (Supplementary Table S2).

**Table 3:** Pre- and post-ablation heart rate variability analysis according to the AFCA modality.

	Pre-IPTW				Post-IPTW			
	Cryo-PVI	HPSD-PVI	Conventional-PVI	*p*-Value	Cryo-PVI	HPSD-PVI	Conventional-PVI	*p*-Value
Total, *n*	73	136	1,049		54^a^	85	951	
Pre-ablation HRV								
Mean HR, bpm	63 (58–70)	67 (58–76)	66 (60–74)	0.141	63 (58–71)	68 (61–76)	66 (60–74)	0.230
rMSSD, ms	25.0 (20.0–30.0)	24.0 (18.0–33.0)	26.0 (18.0–34.0)	0.805	24.6 (21.0–30.0)	23.2 (17.2–30.0)	26.0 (18.0–34.0)	0.381
LF, Hz	13.2 (10.4–19.9)	14.8 (7.9–21.4)	14.0 (8.7–20.2)	0.609	14.2 (11.1–19.8)	13.5 (9.0–20.3)	13.8 (8.6–19.8)	0.268
HF, Hz	9.6 (6.8–12.3)	9.3 (6.1–13.6)	8.9 (6.2–12.9)	0.757	9.8 (7.1–11.9)	9.3 (6.1–12.2)	8.9 (6.2–12.8)	0.446
LF/HF ratio	1.56 (1.30–1.94)	1.55 (1.01–1.85)	1.50 (1.17–1.87)	0.511	1.52 (1.32–1.97)	1.58 (1.04–1.85)	1.50 (1.16–1.86)	0.434
Three-month post-ablation HRV								
Mean HR, bpm	71 (61–79)	73 (66–81)	73 (66–80)	0.273	71 (61–79)	73 (66–81)	73 (66–80)	0.618
rMSSD, ms	16.0 (13.0–22.0)	15.0 (11.0–24.0)	15.0 (11.0–23.0)	0.311	17.0 (13.0–21.7)	15.0 (12.0–24.0)	15.0 (11.0–23.0)	0.227
LF, Hz	7.4 (5.4–10.7)	6.7 (3.3–11.0)	6.2 (3.6–10.9)	0.001	7.5 (5.4–10.7)	8.5 (4.0–11.4)	6.1 (3.5–10.8)	0.009
HF, Hz	5.8 (4.6–10.0)	5.5 (3.8–9.3)	5.4 (3.9–8.5)	0.205	5.9 (4.5–8.3)	5.8 (4.1–9.3)	5.4 (3.9–8.6)	0.322
LF/HF ratio	1.20 (0.98–1.63)	1.11 (0.83–1.45)	1.07 (0.78–1.47)	0.018	1.23 (0.98–1.65)	1.19 (0.9–1.65)	1.06 (0.78–1.42)	0.006
One-year post-ablation HRV								
Mean HR, bpm	71 (64–78)	72 (66–79)	73 (66–80)	0.300	72 (65–79)	72 (67–80)	73 (66–80)	0.569
rMSSD, ms	17.0 (13.3–21.0)	16.0 (12.0–25.0)	15.0 (11.0–23.0)	0.196	16.2 (14.0–21.0)	16.0 (11.5–25.0)	15.0 (11.0–23.0)	0.339
LF, Hz	9.9 (7.5–12.5)	8.7 (5.4–12.8)	7.6 (4.9–12.0)	<0.001	10.2 (7.8–12.7)	9.1 (6.3–14.2)	7.6 (4.9–12.0)	<0.001
HF, Hz	6.2 (5.0–8.4)	5.7 (4.3–9.0)	5.7 (4.1–8.5)	0.129	5.9 (4.9–7.8)	6.4 (4.3–10.0)	5.6 (4.1–8.5)	0.244
LF/HF ratio	1.52 (1.22–1.95)	1.32 (1.04–1.65)	1.29 (1.00–1.67)	<0.001	1.59 (1.26–1.99)	1.28 (0.99–1.66)	1.40 (1.06–1.82)	<0.001

HRV, heart rate variability; HR, heart rate; rMSSD, root mean square of successive differences; LF, low frequency; HF, high frequency. Other abbreviations are the same as in Table 1.

^a^ Effective sample size.

The results for risk of clinical recurrence according to post-ablation HRV parameters are provided in Supplementary Table S3. Among the HRV parameters, high LF and increased LF (delta LF) after AFCA were associated with an increased risk of clinical recurrence.

## Discussion

### Major findings

In this propensity score-weighted, retrospective analysis of a single-center cohort, we compared the efficacy and safety outcomes along with cardiac autonomic nervous activity among AF patients who underwent Cryo-PVI, HPSD-PVI, or conventional-PVI. Similar rhythm outcomes and complication rates among the three ablation groups were reported. However, Cryo-PVI was associated with better rhythm outcomes in patients with PAF but worse rhythm outcomes inpatients with PeAF compared with conventional-PVI. In addition, Cryo-PVI was associated with better rhythm outcomes among patients with an LAVI of <34 mL/m^2^ compared with HPSD-PVI and conventional-PVI. We found significantly shorter ablation and procedure times but higher cardiac sympathetic nervous activity over long-term periods in the Cryo-PVI group compared with the HPSD-PVI or conventional-PVI group.

### AF types and the PVI methodology

Although randomized trials on the efficacy of Cryo-PVI vs. conventional-PVI have reported similar AF recurrences in PAF (20, 21), several studies consistently suggested fewer PV reconnections among patients who underwent Cryo-PVI (22, 23). However, the results of these randomized studies were based on a small number of patients that did not compare the rhythm outcomes among patients with PeAF (24, 25). In terms of PeAF, several case-only comparison studies have reported similar AF recurrences between the Cryo-PVI and conventional-PVI (26–28), and one study group reported that Cryo-PVI had a worse 1-year outcome than conventional-PVI (29).

Our study suggests that patients with PAF may benefit more from Cryo-PVI than from conventional-PVI in contrast to patients with PeAF. The reason for this is not clear, but PV ostium remodeling (diameter and shape) in patients with PeAF might have resulted in an imperfect cryoballoon tissue contact, or the cryothermal energy transfer could have been affected by the degree of tissue fibrosis (30–32). This possibility is supported by better rhythm outcomes among patients with an LAVI of <34 mL/m^2^ in the Cryo-PVI group compared with the HPSD-PVI or conventional-PVI group.

Martino et al. (33) reported that stepwise additional ablation including endocardial and epicardial LA ablation after PVI showed favorable rhythm outcomes in the patients with non-paroxysmal AF. Because the current study excluded patients who underwent extra-PV LA ablation and showed differing results in rhythm outcomes according to the AF type, future research on the effect of additional LA ablation after both Cryo-PVI and RF-PVI in patients with persistent AF might benefit.

### Cardiac autonomic nervous activity after AF ablation

The cardiac autonomic nervous system is reported to initiate and maintain AF, and autonomic neural modulation via a circumferential PVI and SVC-RA ablation is reported to reduce AF recurrence (34–36). The post-procedural HRV analysis in this study suggests that Cryo-PVI is associated with a higher cardiac sympathetic nervous activity and sympatho-vagal balance compared with HPSD-PVI or conventional-PVI. Cryo-PVI may generate large tissue damage due to a larger balloon–tissue contact area compared with conventional-PVI that may result in sympathetic/parasympathetic nerve sprouting that leads to an overall higher post-procedural sympathetic nervous activity and sympatho-vagal balance, especially among patients with PeAF (37, 38). HPSD-PVI had the lowest LF/HF ratio at 1-year post-procedure, suggesting an optimal cardiac autonomic neural modulation but equivalent long-term rhythm outcome.

The clinical implications of post-AFCA HRVs have been described in previous studies. Kang et al. (39) reported that reduced LF/HF ratio after 3 months of AFCA was associated with poor rhythm outcomes. Yu et al. (40) reported that high sinus heart rate after AFCA was associated with decreased LF and better rhythm outcomes. In this study, higher 3-month and 1-year post-ablation sympathetic nervous activity (LF) was associated with an increased risk of clinical recurrence, and patients who underwent Cryo-PVI had higher sympathetic nervous activity and higher sympatho-vagal balance at post-ablation periods. These findings showed that the patients who had increased HRV parameters such as LF and LF/HF ratio after Cryo-PVI, representing high sympathetic activity and sympatho-vagal balance, might be at high risk for clinical recurrence.

### Future technologies in AF ablation

Cryo-PVI ablates a large volume of PV ostium at a single shot that involves less catheter manipulation and shorter procedural time compared with RF-PVI. Although Cryo-PVI is associated with higher radiation exposure initially to identify proper PV occlusion using radiation and contrast injection, intracardiac echocardiography-guided zero-fluoroscopy Cryo-PVI showed comparable rhythm outcomes in a recent clinical trial (41). In contrast, RF-PVI ablates PV ostium in a point-to-point manner and requires more catheter manipulation which depends on the operator's skills and experiences. Steerable sheaths help the operators improve contact and stability of the ablation catheter, leading to achieve efficient lesion formation for AF ablation. Recently, with the advent of visualizable steerable sheaths, RF-PVI is associated with minimal radiation exposure (42, 43), and zero-fluoroscopy RF-PVI (44) showed equal efficacy and safety compared with traditional procedures (45). Therefore, future direction for AF ablation using cryoballoon and radiofrequency catheter possibly studies better rhythm outcomes, lower complication rates, reduced procedure time, and also reduced radiation exposure.

### Limitations

This study has several potential limitations. First, this was a single-center study from a tertiary hospital, and the study sample may not represent the general AF patients, in which the results of this study need careful interpretation and further multi-center studies would benefit. However, data from a single center are still valuable because the AFCA procedure as well as the rhythm follow-up protocols were largely consistent. Second, the HRV analysis in this study should be interpreted with caution because not all patients had available post-procedural HRV parameters. However, our results are one of the largest single-center, long-term pre- and post-ablation HRV analysis (46) that might provide a reasonable estimation. In addition, the complementary analysis (Supplementary Table S2) that included all patients who underwent HRV at pre- and post-ablation at 3 months, 1 year, and 2 years might provide an additional insight.

Third, despite appropriate efforts to perform unbiased comparisons between the AFCA groups using propensity score-weighted models, a residual confounding from other potential covariates such as AF duration or AF burden, which were not accounted for in this study, remains. In addition, because of the retrospective, observational nature of the study, a causal relationship cannot be drawn, and further randomized trials are needed.

Fourth, this study included patients who received right atrial ablation, and a slight difference in the number of right atrial ablations performed among the three ablation groups was found. Although we performed the doubly robust IPTW model to reduce confounding, concerns for residual confounding remains, and the results of this study requires careful interpretation.

## Conclusions

Cryo-PVI, HPSD-PVI, and conventional-PVI had similar rhythm outcomes and complication rates in patients with AF. However, Cryo-PVI was associated with better rhythm outcomes in patients with PAF but worse rhythm outcomes in patients with PeAF compared with conventional-PVI. Cryo-PVI had significantly shorter procedure times but had higher long-term post-procedural sympathetic nervous activity. AF type might be useful information for the selection of AFCA modality for better rhythm outcomes in patients with AF.

## Data Availability

The raw data supporting the conclusions of this article will be made available by the authors, without undue reservation.
