# Supplementary material for: Comparison of pulmonary vein isolation using cryoballoon, high-power short-duration, and conventional radiofrequency ablation for atrial fibrillation: a propensity score-weighted study
Source: Front Cardiovasc Med. 2023 Oct 9;10:1238363. doi: 10.3389/fcvm.2023.1238363 (PMC10590885; doi:10.3389/fcvm.2023.1238363)
Supplement: Supplementary file 1 [file Table1.docx]

**Supplementary Materials**

**Supplementary Table 1.** Cox regression analysis of the risk of clinical recurrence after AFCA.

**Supplementary Table 2.** Pre- and post-ablation heart rate variability analysis according to AFCA modality among participants without serial HRV follow-up data.

**Supplementary Table 3**. Risk of clinical recurrence according to 3-month and 1-year post-ablation HRV.

**Supplementary Figure 1.** Absolute standardized differences for baseline covariates before and after inverse probability of treatment weight.

**Supplementary Figure 2**. Distribution of propensity score before and after inverse probability of treatment weight.

**Supplementary Figure 3**. Sensitivity analysis for the risk of clinical recurrence after excluding patients who underwent conventional-PVI during earlier years than Cryo or HPSD-PVI.

**Supplementary Figure 4**. Sensitivity analysis for the risk of clinical recurrence after excluding patients who underwent AFCA with non-contact force electrode catheters.

**Supplementary Table 1.** Cox regression analysis of the risk of clinical recurrence after AFCA.

|  | **Unweighted HR (95% CI)** | **p-value** | **Weighted HR^a^**  **(95% CI)** | **p-value** |
| --- | --- | --- | --- | --- |
| **Ablation method** |  |  |  |  |
| Conventional-PVI | Reference |  | Reference | - |
| Cryo-PVI | 1.26 (1.05-1.52) | 0.015 | 0.90 (0.73-1.11) | 0.321 |
| HPSD-PVI | 0.98 (0.81-1.19) | 0.849 | 0.96 (0.75-1.21) | 0.711 |
| **Paroxysmal AF** | 0.52 (0.45-0.61) | <0.001 | 0.47 (0.39-0.56) | <0.001 |
| **Age** | 1.00 (0.99-1.01) | 0.339 | 1.00 (0.99-1.01) | 0.284 |
| **Men** | 0.99 (0.85-1.17) | 0.977 | 0.93 (0.75-1.14) | 0.481 |
| **Height** | 1.01 (0.45-2.29) | 0.971 | 0.56 (0.19-1.67) | 0.298 |
| **LVEF** | 0.99 (0.98-1.00) | 0.004 | 1.00 (0.99-1.01) | 0.717 |
| **E/e’** | 1.00 (0.98-1.02) | 0.855 | 1.01 (0.99-1.04) | 0.339 |
| **LAVI** | 1.02 (1.01-1.03) | <0.001 | 1.02 (1.01-1.03) | <0.001 |
| **LVEDD** | 1.00 (0.99-1.02) | 0.730 | 0.99 (0.97-1.01) | 0.450 |
| **HTN** | 1.08 (0.93-1.24) | 0.318 | 1.02 (0.85-1.23) | 0.799 |
| **DM** | 1.05 (0.87-1.28) | 0.598 | 1.01 (0.78-1.29) | 0.964 |
| **BMI** | 1.01 (0.99-1.04) | 0.235 | 1.02 (0.99-1.05) | 0.240 |
| **Heart failure** | 1.26 (1.03-1.55) | 0.025 | 0.97 (0.75-1.24) | 0.790 |
| **Vascular disease** | 0.80 (0.59-1.07) | 0.130 | 0.92 (0.61-1.39) | 0.692 |
| **CHA_2_DS_2_VASc** | 1.04 (0.99-1.09) | 0.166 | 1.04 (0.97-1.10) | 0.273 |
| **H2FPEF** | 1.02 (0.97-1.07) | 0.468 | 1.03 (0.96-1.10) | 0.393 |
| **SVC-RA ablation** | 0.60 (0.52-0.69) | <0.001 | 0.47 (0.39-0.56) | <0.001 |

HR, hazard ratio; CI, confidence interval. Other abbreviations are the same as in Table 1.

^a^Weighted HR was additionally adjusted for AF type and SVC-RA ablation.

**Supplementary Table 2.** Pre- and post-ablation heart rate variability analysis according to AFCA modality among participants without serial HRV follow-up data.

|  | **Pre-IPTW** | | | | **Post-IPTW** | | | |
| --- | --- | --- | --- | --- | --- | --- | --- | --- |
|  | **Cryo-PVI** | **HPSD-PVI** | **Conventional-PVI** | **p-value** | **Cryo-PVI** | **HPSD-PVI** | **Conventional-PVI** | **p-value** |
| **Pre-ablation HRV (n=1,529)** | | | | | | | | |
| mean HR, bpm | 66 (59-73) | 67 (61-75) | 67 (61-75) | 0.146 | 67 (59-73) | 67 (61-76) | 67 (61-76) | 0.199 |
| rMSSD, ms | 23.0 (16.0-31.0) | 25.0 (17.0-34.0) | 24.0 (17.0-33.0) | 0.122 | 24.0 (17.3-32.0) | 25.9 (16.6-35.0) | 24.0 (17.0-33.0) | 0.966 |
| LF, Hz | 12.4 (7.0-19.0) | 13.7 (8.3-20.5) | 13.5 (8.4-19.7) | 0.088 | 13.4 (8.5-19.6) | 13.1 (8.8-20.1) | 13.1 (8.1-19.5) | 0.973 |
| HF, Hz | 8.0 (5.2-12.1) | 9.1 (5.9-13.8) | 8.6 (6.0-12.5) | 0.238 | 8.4 (5.9-12.9) | 8.8 (5.4-13.6) | 8.5 (5.9-12.5) | 0.917 |
| LF/HF ratio | 1.36 (0.97-1.77) | 1.51 (1.07-1.86) | 1.52 (1.17-1.88) | 0.004 | 1.50 (1.09-1.82) | 1.54 (1.09-1.88) | 1.50 (1.14-1.87) | 0.710 |
| **Three-month post-ablation HRV (n=1,809)** | | | | | | | | |
| mean HR, bpm | 72 (65-79) | 73 (67-81) | 72 (66-80) | 0.022 | 72 (66-79) | 72 (65-80) | 72 (65-80) | 0.961 |
| rMSSD, ms | 15.0 (12.0-20.3) | 16.0 (12.0-22.0) | 15.0 (11.0-23.0) | 0.483 | 16.0 (12.0-19.0) | 16.0 (12.0-23.0) | 15.0 (11.0-23.0) | 0.481 |
| LF, Hz | 7.1 (4.9-11.4) | 6.4 (3.6-10.9) | 6.1 (3.5-11.0) | 0.016 | 7.3 (5.0-11.5) | 7.1 (3.9-11.4) | 6.0 (3.4-10.9) | 0.003 |
| HF, Hz | 5.6 (4.3-7.4) | 5.6 (4.0-8.3) | 5.4 (3.9-8.4) | 0.727 | 5.7 (4.1-7.4) | 5.7 (4.0-8.2) | 5.4 (3.9-8.5) | 0.630 |
| LF/HF ratio | 1.22 (0.95-1.61) | 1.04 (0.81-1.44) | 1.08 (0.78-1.47) | 0.001 | 1.30 (0.97-1.62) | 1.15 (0.85-1.61) | 1.06 (0.77-1.43) | <0.001 |
| **One-year post-ablation HRV (n=1,464)** | | | | | | | | |
| mean HR, bpm | 70 (64-77) | 71 (66-78) | 73 (66-80) | 0.001 | 71 (65-78) | 70 (65-77) | 73 (66-80) | 0.009 |
| rMSSD, ms | 17.0 (14.0-23.3) | 15.0 (11.0-23.5) | 15.0 (12.0-23.0) | 0.061 | 16.0 (14.0-21.2) | 16.0 (11.5-24.0) | 15.0 (12.0-24.0) | 0.244 |
| LF, Hz | 9.7 (7.1-12.4) | 8.4 (5.3-12.1) | 7.7 (5.0-12.0) | 0.001 | 9.8 (7.2-12.5) | 8.9 (6.0-13.0) | 7.7 (4.9-12.0) | <0.001 |
| HF, Hz | 6.2 (4.8-8.8) | 5.6 (4.3-8.7) | 5.7 (4.1-8.5) | 0.106 | 6.2 (4.8-8.0) | 5.7 (4.3-9.0) | 5.7 (4.1-8.5) | 0.241 |
| LF/HF ratio | 1.44 (1.14-1.89) | 1.31 (1.02-1.66) | 1.29 (1.00-1.68) | 0.001 | 1.53 (1.20-1.95) | 1.28 (0.99-1.66) | 1.40 (1.05-1.82) | <0.001 |
| **Two-year post-ablation HRV (n=637)** | | | | | | | | |
| mean HR, bpm | 69 (63-75) | 68 (63-75) | 72 (65-78) | 0.017 | 70 (63-75) | 67 (61-74) | 72 (65-78) | 0.007 |
| rMSSD, ms | 17.5 (15.0-27.5) | 16.0 (13.0-27.0) | 15.0 (12.0-23.0) | 0.006 | 19.5 (15.0-26.9) | 16.0 (13.0-27.8) | 15.0 (12.0-23.0) | 0.005 |
| LF, Hz | 9.8 (8.1-13.0) | 8.4 (6.0-13.1) | 8.1 (5.3-12.1) | <0.001 | 10.1 (8.8-13.2) | 8.7 (6.0-13.4) | 8.0 (5.3-12.1) | <0.001 |
| HF, Hz | 6.8 (5.3-10.3) | 6.2 (4.5-9.8) | 5.6 (4.1-8.1) | 0.002 | 8.1 (5.4-10.3) | 6.2 (4.5-10.2) | 5.6 (4.1-8.2) | 0.001 |
| LF/HF ratio | 1.43 (1.09-1.79) | 1.21 (0.91-1.60) | 1.37 (1.03-1.73) | 0.006 | 1.45 (1.09-1.70) | 1.18 (0.92-1.56) | 1.34 (1.02-1.71) | 0.001 |

HRV, heart rate variability; HR, heart rate; rMSSD, root mean square of successive differences; LF, low frequency; HF, high frequency. Other abbreviations are the same as in Table 1.

**Supplementary Table 3**. Risk of clinical recurrence according to 3-month and 1-year post-ablation HRV.

|  | **Unweighted HR per 1-SD increase** | | **Weighted HR per 1-SD increase** | |
| --- | --- | --- | --- | --- |
| **3-month post-ablation HRV** | HR (95% CI) | P-value | HR (95% CI) | P-value |
| Mean heart rate^a^ | 0.90 (0.81-1.00) | 0.058 | 0.93 (0.83-1.05) | 0.163 |
| rMSSD | 1.16 (1.07-1.26) | <0.001 | 1.11 (0.97-1.27) | 0.133 |
| LF | 1.13 (1.05-1.21) | <0.001 | 1.11 (1.01-1.21) | 0.031 |
| HF | 1.12 (1.03-1.21) | 0.008 | 1.11 (0.99-1.23) | 0.064 |
| LF/HF ratio | 1.01 (0.92-1.12) | 0.815 | 1.05 (0.94-1.14) | 0.732 |
| **1-year post-ablation HRV^a^** |  |  |  |  |
| Mean heart rate^a^ | 1.02 (0.84-1.22) | 0.871 | 1.08 (0.85-1.37) | 0.510 |
| rMSSD | 1.15 (0.98-1.34) | 0.085 | 1.14 (0.95-1.38) | 0.164 |
| LF | 1.20 (1.07-1.34) | 0.002 | 1.20 (1.05-1.37) | 0.008 |
| HF | 1.16 (1.01-1.33) | 0.043 | 1.16 (0.98-1.36) | 0.083 |
| LF/HF ratio | 1.05 (0.88-1.26) | 0.595 | 1.00 (0.79-1.28) | 0.971 |
| **HRV change (3 month post ablation-pre ablation)** |  |  |  |  |
| Delta mean HR | 0.90 (0.79-1.01) | 0.079 | 1.11 (0.89-1.40) | 0.345 |
| Delta rMSSD | 1.15 (1.02-1.30) | 0.028 | 1.08 (0.94-1.25) | 0.282 |
| Delta LF | 1.98 (1.42-2.77) | <0.001 | 1.66 (1.10-2.51) | 0.015 |
| Delta HF | 1.18 (1.04-1.34) | 0.010 | 1.11 (0.96-1.29) | 0.159 |
| Delta LF/HF | 1.20 (1.02-1.44) | 0.031 | 1.14 (0.95-1.38) | 0.163 |
| **HRV change (1 year post ablation-pre ablation)** |  |  |  |  |
| Delta mean HR | 0.88 (0.68-1.13) | 0.300 | 0.88 (0.68-1.14) | 0.349 |
| Delta rMSSD | 0.90 (0.70-1.15) | 0.388 | 0.90 (0.68-1.19) | 0.462 |
| Delta LF | 2.60 (1.18-7.21) | <0.001 | 2.32 (1.01-5.36) | 0.049 |
| Delta HF | 1.19 (0.93-1.52) | 0.174 | 1.18 (0.97-1.45) | 0.099 |
| Delta LF/HF | 1.05 (0.81-1.36) | 0.694 | 1.02 (0.83-1.25) | 0.880 |

^a^The risk of clinical recurrence according to 1-year post-ablation HRV was calculated only for AF recurrences that occurred after 1-year

**Supplementary Figure 1.** Absolute standardized differences for baseline covariates before and after inverse probability of treatment weight.

**
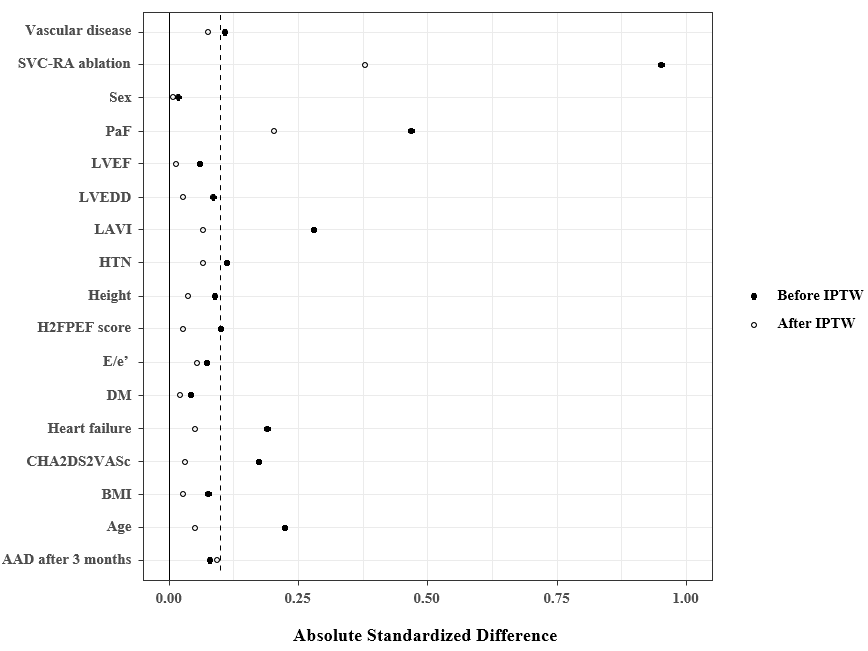
**

Abbreviations are the same as Table 1. Weights were truncated at 1^th^ and 99^th^ percentile.

**Supplementary Figure 2**. Distribution of propensity score before and after inverse probability of treatment weight.

**
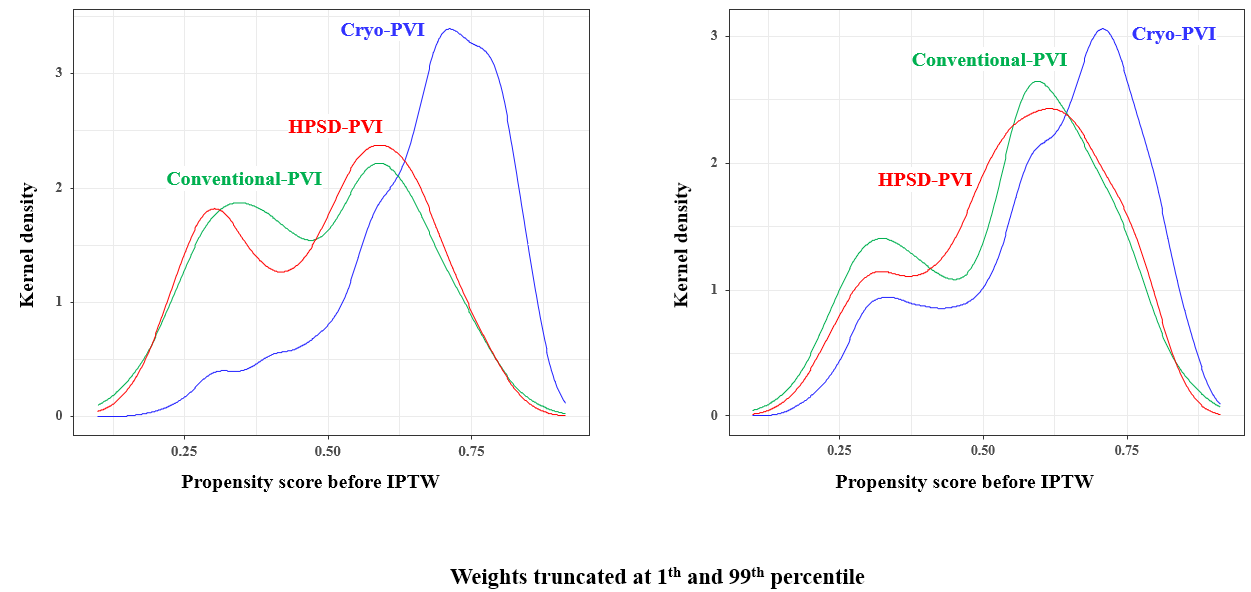
**

Abbreviations are the same as Table 1.

**Supplementary Figure 3**. Sensitivity analysis for the risk of clinical recurrence after excluding patients who underwent conventional-PVI during earlier years than Cryo or HPSD-PVI.


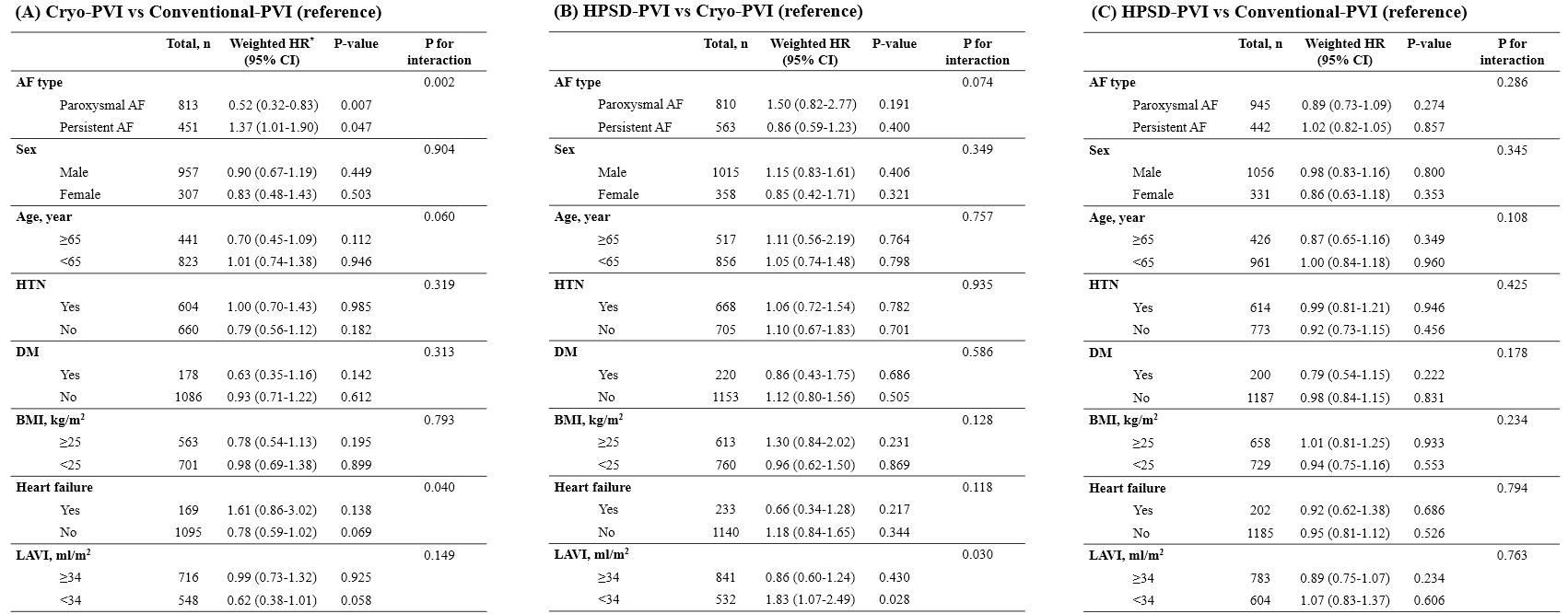


Abbreviations are the same as in Table 1.

^a^Weighted HR was additionally adjusted for AF type and SVC-RA ablation.

**Supplementary Figure 4**. Sensitivity analysis for the risk of clinical recurrence after excluding patients who underwent AFCA with non-contact force electrode catheters.


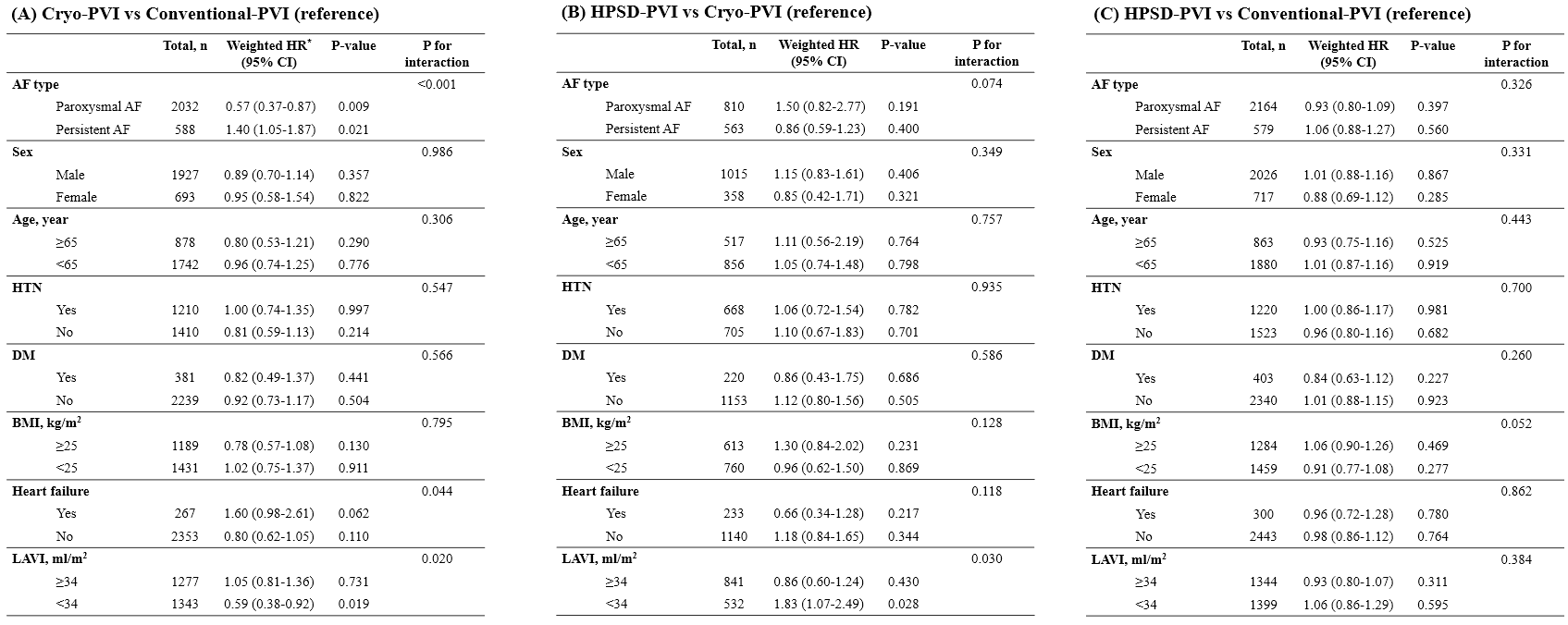


Abbreviations are the same as in Table 1.

^a^Weighted HR was additionally adjusted for AF type and SVC-RA ablation.
